# Supplementary figures and images for: Saliva sampling method influences oral microbiome composition and taxa distribution associated with oral diseases
Source: PLoS One. 2024 Mar 28;19(3):e0301016. doi: 10.1371/journal.pone.0301016 (PMC10977688; doi:10.1371/journal.pone.0301016)

**Figure S1**

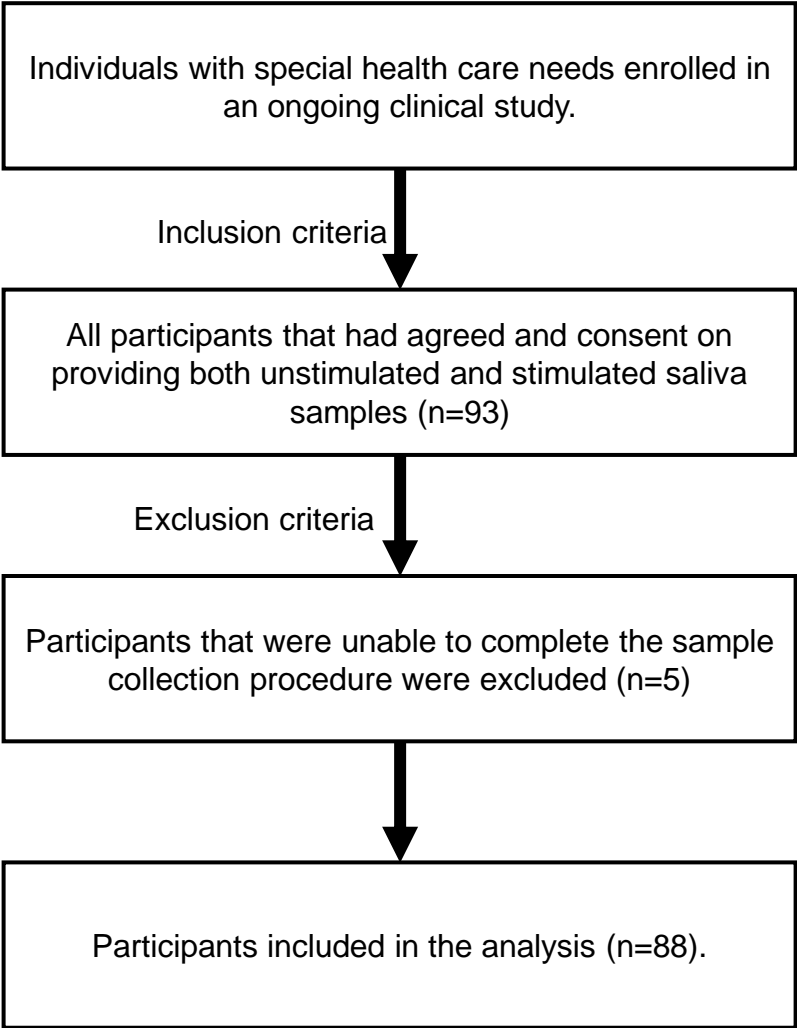

Supplement: S1 Fig — After the selection based on our inclusion and exclusion criteria, 88 participants provided stimulated and unstimulated saliva samples. (PDF) [file pone.0301016.s001.pdf]

Figure S2

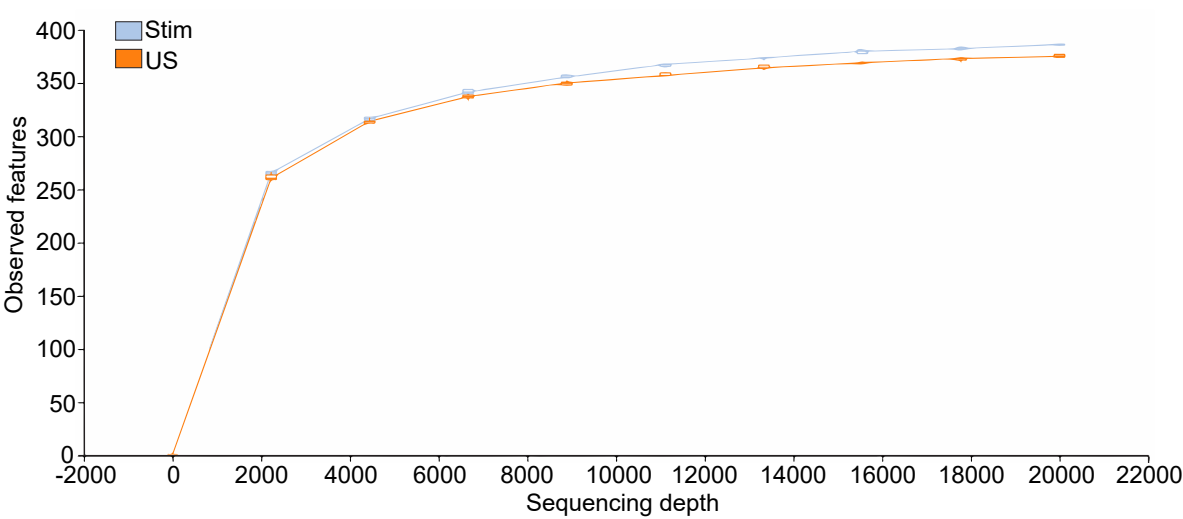

Supplement: S2 Fig — (PDF) [file pone.0301016.s002.pdf]

Figure S3

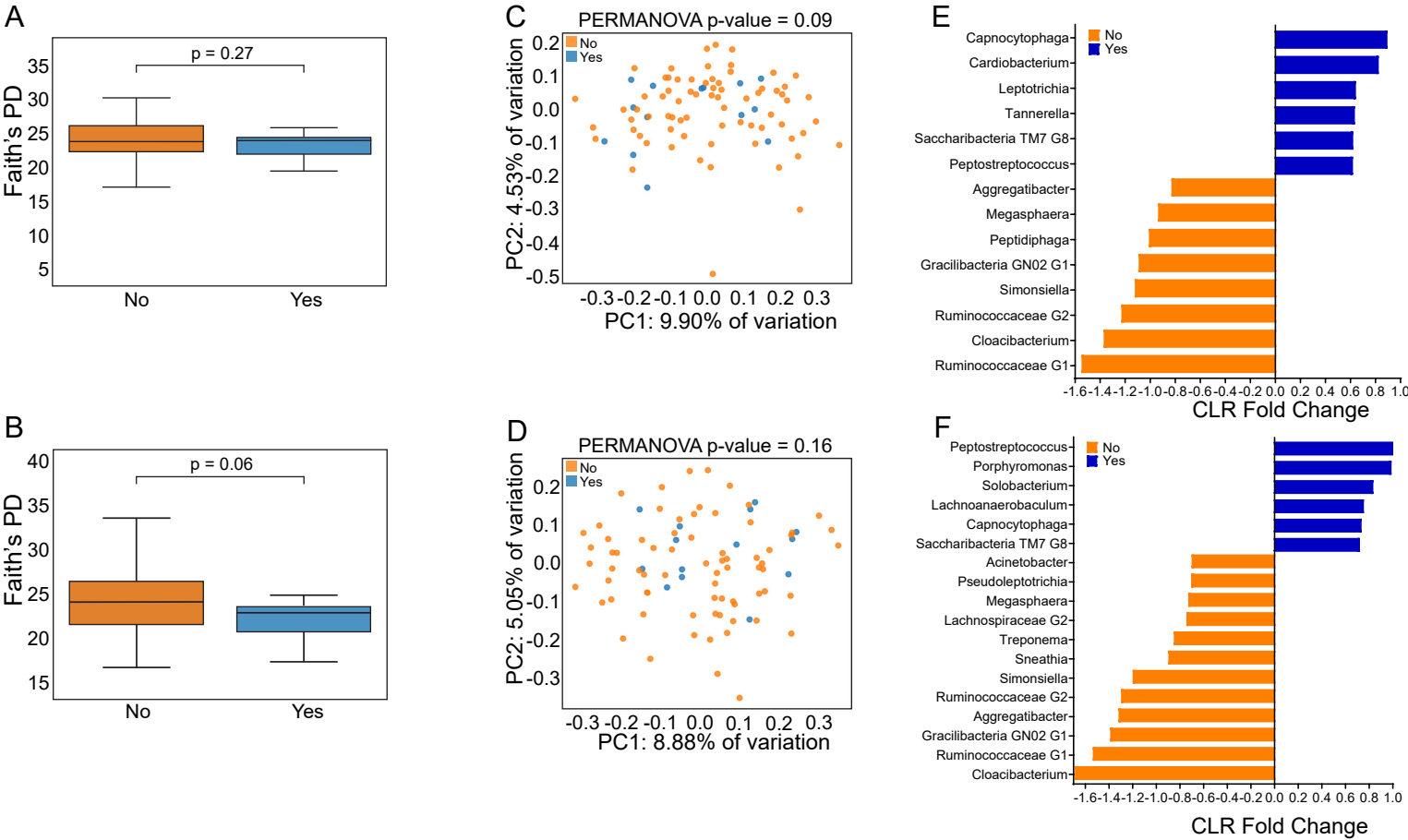

Supplement: S3 Fig — Alpha diversity (Faith’s PD) comparison of microbiome in Stim (A) and US (B). Principal components plot and PERMANOVA beta diversity analysis comparing Stim (C) and US (D) among participants with and without reported antibiotic use. Beta diversity centered log ratio (CLR) fold change showing differential enrichment of taxa at the genus level between Stim (E) and US (F). (PDF) [file pone.0301016.s003.pdf]

Figure S4

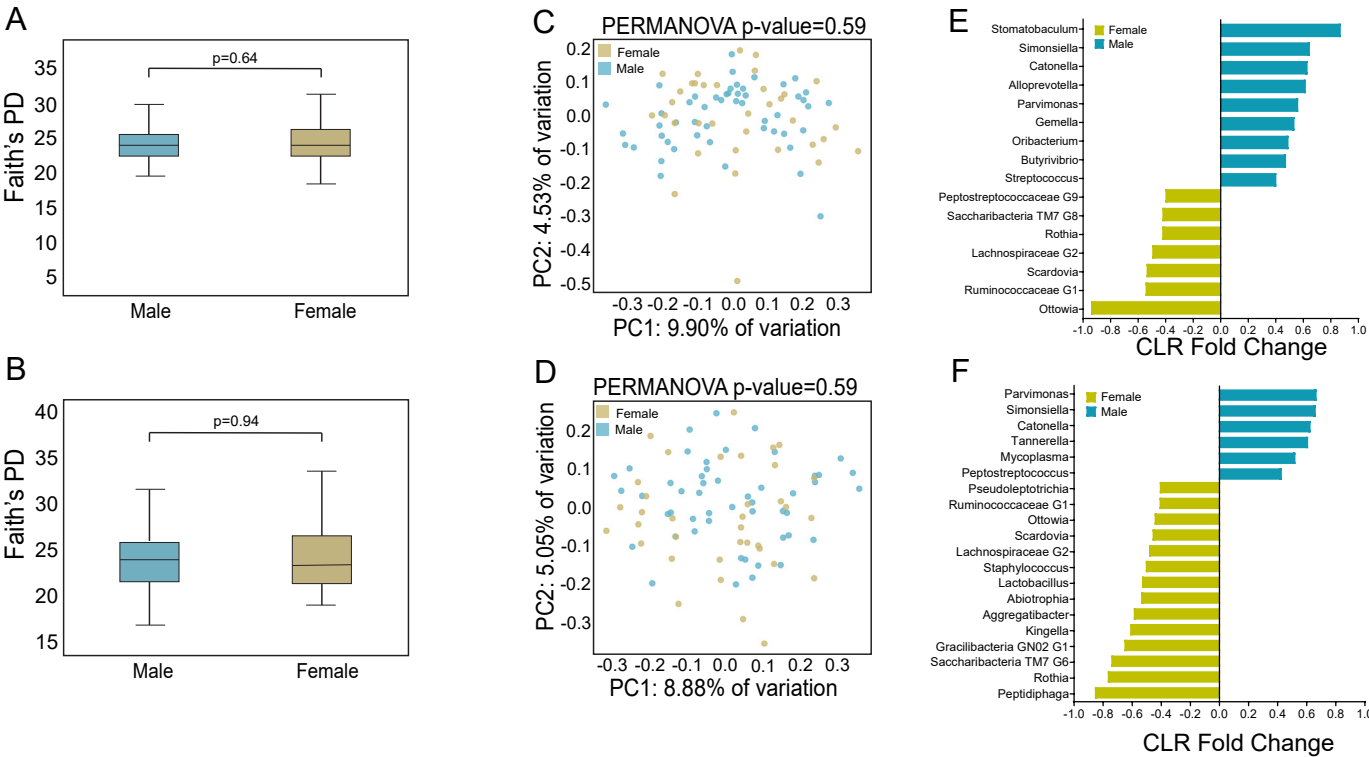

Supplement: S4 Fig — Alpha diversity (Faith’s PD) comparison of microbial composition in Stim (A) and US (B). Principal components plot comparing Stim (C) and US (D) based on reported sex, PERMANOVA beta diversity test. Beta diversity centered log ratio (CLR) fold change showing differential enrichment of taxa at the genus level between Stim (E) and US (F). (PDF) [file pone.0301016.s004.pdf]

Figure S5

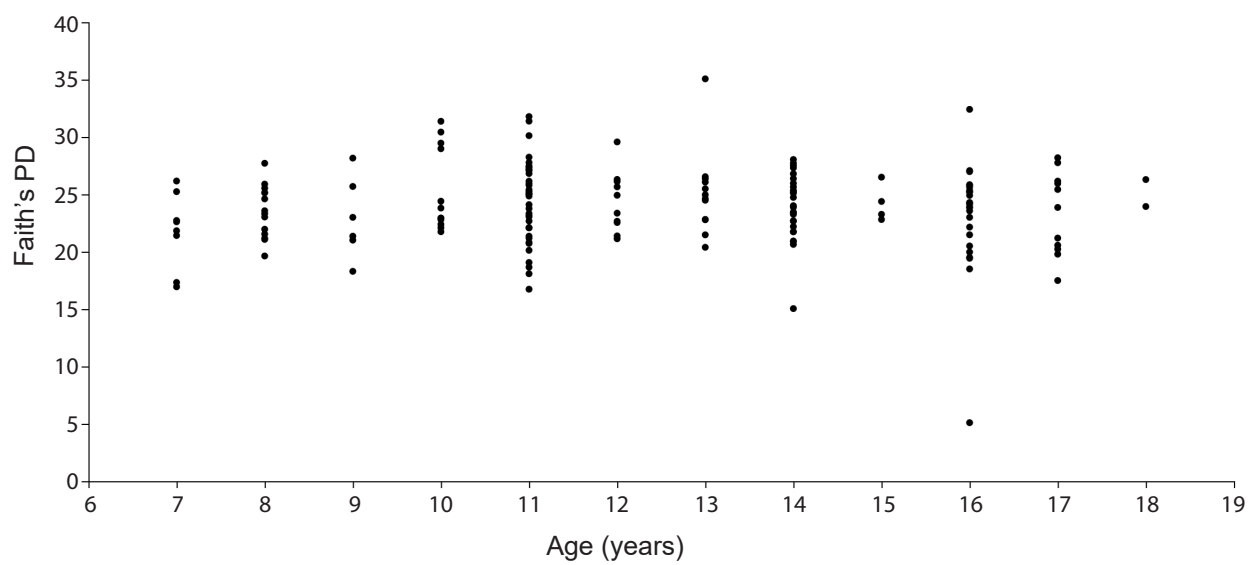

Supplement: S5 Fig — No significant differences were found using Spearman correlation (p = 0.67). (PDF) [file pone.0301016.s005.pdf]

Figure S6

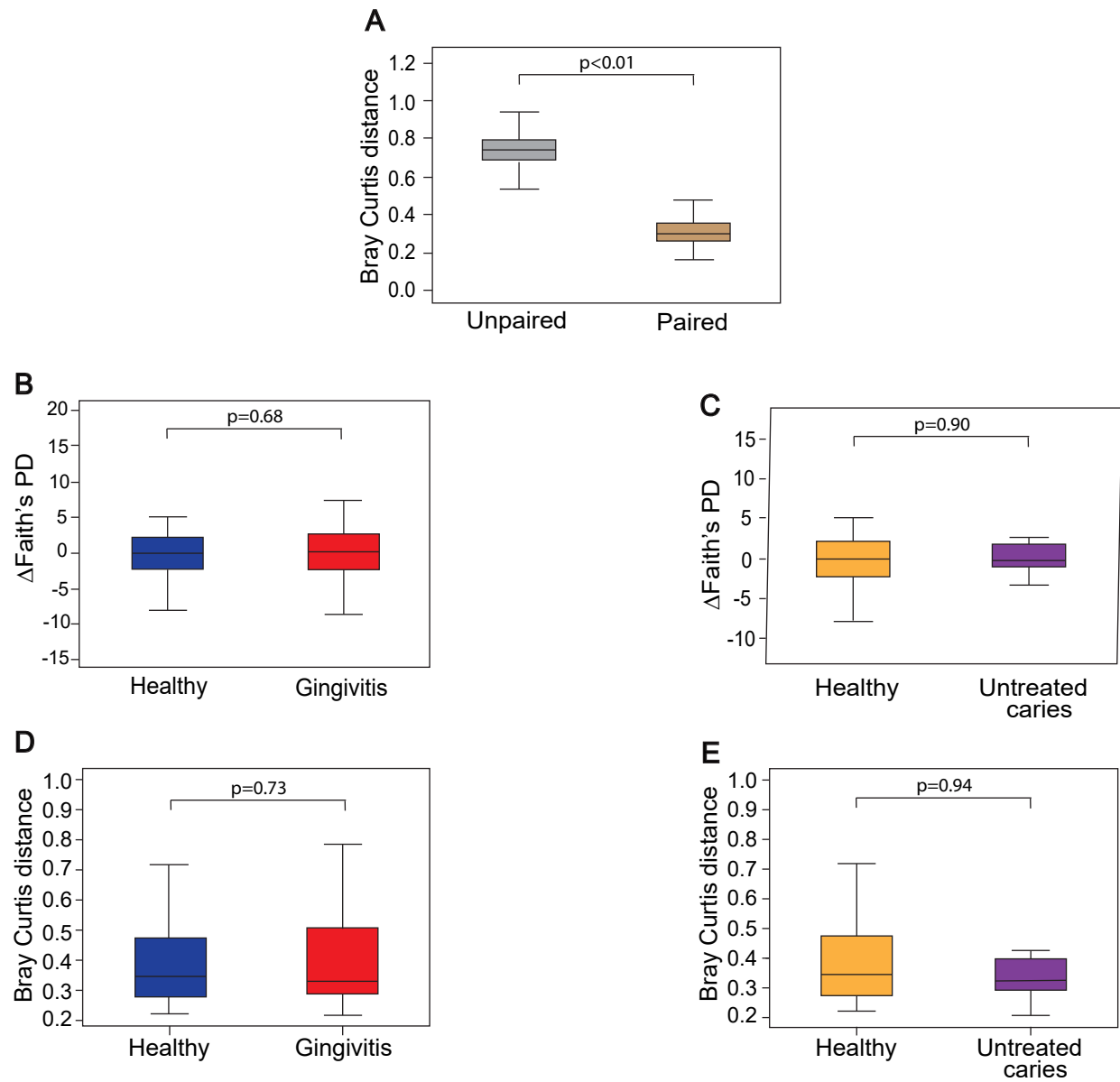

Supplement: S6 Fig — A) Bray Curtis Beta diversity distance of microbial composition within subjects (paired) as compared to across subjects (unpaired). Pairwise Faith’s PD difference of microbial composition between stimulated and unstimulated samples by gingivitis status (B) and untreated caries (C). Pairwise Bray Curtis Beta diversity distance difference of microbial composition between stimulated and unstimulated samples by gingivitis status (D) and untreated caries (E). (PDF) [file pone.0301016.s006.pdf]

Figure S7

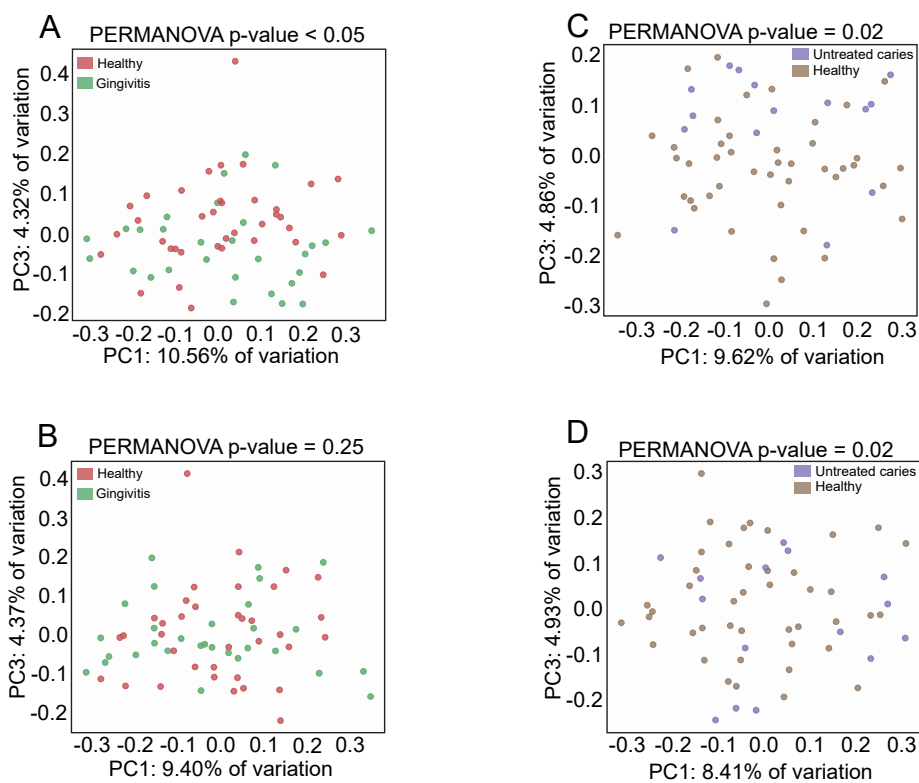

Supplement: S7 Fig — Principal components plots using PC1 and PC3 comparing subjects with gingivitis versus healthy controls in both Stim (A) and US (B) saliva, and plots comparing subjects with untreated caries versus healthy controls in both Stim (C) and US (D) saliva. PERMANOVA beta diversity analysis indicates significant difference (p<0.05) in taxonomic composition. (PDF) [file pone.0301016.s007.pdf]
