# Supplementary material for: Saliva sampling method influences oral microbiome composition and taxa distribution associated with oral diseases
Source: PLoS One. 2024 Mar 28;19(3):e0301016. doi: 10.1371/journal.pone.0301016 (PMC10977688; doi:10.1371/journal.pone.0301016)
Supplement: Attachment — Submitted filename: Response to Reviewers.docx [file pone.0301016.s008.docx]

We would like to thank the editor and reviewer for their insightful comments and valuable suggestions. We have addressed all comments and believe that it has resulted in a more concise and improved manuscript.

Journal requirements:

When submitting your revision, we need you to address these additional requirements.

1. Please ensure that your manuscript meets PLOS ONE's style requirements, including those for file naming. The PLOS ONE style templates can be found at https://journals.plos.org/plosone/s/file?id=wjVg/PLOSOne_formatting_sample_main_body.pdf and https://journals.plos.org/plosone/s/file?id=ba62/PLOSOne_formatting_sample_title_authors_affiliations.pdf

The manuscript has been re-organized to comply with the journal requirements for the main text and the authors affiliations.

2. Please provide additional details regarding participant consent. In the ethics statement in the Methods and online submission information, please ensure that you have specified what type you obtained (for instance, written or verbal, and if verbal, how it was documented and witnessed).

Additional details on the ethics statement were added in the Methods so that it clarifies whether a written or verbal consent/assent was obtained from the study participants (lines 108-115).

3. Thank you for stating the following financial disclosure:

“C.R., D.L.C and M.C.W. were funded in part by support from the National Institute of Dental and Craniofacial Research, NIH (U01DE030418), and J.R.W. was funded by NIH K01 DK119582.” Please state what role the funders took in the study. If the funders had no role, please state: "The funders had no role in study design, data collection and analysis, decision to publish, or preparation of the manuscript." If this statement is not correct you must amend it as needed.

Please include this amended Role of Funder statement in your cover letter; we will change the online submission form on your behalf.

A statement about the role of the funders of the study was added to the author’s contribution section (lines 349-351) and the cover letter.

4. Your ethics statement should only appear in the Methods section of your manuscript. If your ethics statement is written in any section besides the Methods, please move it to the Methods section and delete it from any other section. Please ensure that your ethics statement is included in your manuscript, as the ethics statement entered into the online submission form will not be published alongside your manuscript.

The ethics statement was moved to the Materials and Methods section and deleted from its previous position (lines 108-115).

5. We notice that your supplementary figures are uploaded with the file type 'Figure'. Please amend the file type to 'Supporting Information'. Please ensure that each Supporting Information file has a legend listed in the manuscript after the references list.

The file type was corrected, and a legend of each supporting information figure is now present in the manuscript after references (lines 472-504).

6. Please review your reference list to ensure that it is complete and correct. If you have cited papers that have been retracted, please include the rationale for doing so in the manuscript text, or remove these references and replace them with relevant current references. Any changes to the reference list should be mentioned in the rebuttal letter that accompanies your revised manuscript. If you need to cite a retracted article, indicate the article’s retracted status in the References list and also include a citation and full reference for the retraction notice.

References have been revised. Some references have been added to comply with the reviewer’s comments.

Reviewer #1 (Comments for the Author):

Reviewer #1: The manuscript titled 'Saliva Sampling Method Influences Oral Microbiome Composition and Taxa Distribution Associated with Oral Diseases,' submitted to PLOS ONE, compares the oral microbiome composition using simulated and unstimulated saliva sampling methods. In general, this study is wellconducted, and the figures are good. However, there are many areas that require attention before it can be accepted for publication.

Major comments:

1. Considering the significance of stimulated and unstimulated saliva sampling methods for this paper, the authors are required to provide some background on these methods in the introduction.

The introduction has been amended to now include background information on stimulated and unstimulated saliva samples (lines 70-78).

2. Including a figure illustrating the study design can enhance readers' understanding of the study.

A flow diagram has been added as Figure S1 in the supporting information to clarify the participants selection and study design.

3. The authors are required to provide the accession number for the deposited raw reads.

The accession number for the raw sequences and the metadata have been added to the text (lines 334-338).

Minor comments:

1. The writing for stimulated saliva sampling collection should be clearer in methods.

A clarification for the stimulated saliva collection was included in the methods section (lines 124-127).

2. In lines 155-156, where it is mentioned, 'Taxa were assigned based on matching ASVs to the Human Oral Microbiome database v.15.23 (27),' the authors are advised to provide the criteria used for taxa classification.

Details related to the taxonomic classification were added to the text (lines 172-174).

3. The authors mentioned ANCOM analysis in line 160; they are advised to provide the full name of it and include a citation.

The full name for ANCOM and the respective citation was added to the text (lines 178-179).

4. The authors used PC1 and PC3 for PC plots, whereas it is more common to use PC1 and PC2 for such

plots. It is advised to consider a three-dimensional figure if the authors wish to retain PC3.

Regarding Figure 1, there was a typographical error, and it already presented PC1 and PC2. The error was corrected. All remaining figures were revised to show PC1 and PC2. In addition, the plots for PC1 and PC3 were included as a supplementary figure (Fig. S7).
